# Death of Dr. Harlan

**Published:** 1843-11

**Authors:** 


### DEATH OF DR. HARLAN.

This eminent anatomist and zoologist died in New Orleans suddenly, of apoplexy, in the month of September. Dr. Harlan was in the prime of life, and is a great loss to our profession of which he was an ornament.

